# Differential Response of Potato Toward Inoculation with Taxonomically Diverse Plant Growth Promoting Rhizobacteria

**DOI:** 10.3389/fpls.2016.00144

**Published:** 2016-02-17

**Authors:** Tahir Naqqash, Sohail Hameed, Asma Imran, Muhammad Kashif Hanif, Afshan Majeed, Jan Dirk van Elsas

**Affiliations:** ^1^National Institute for Biotechnology and Genetic EngineeringFaisalabad, Pakistan; ^2^Pakistan Institute of Engineering and Applied SciencesIslamabad, Pakistan; ^3^University of PoonchRawlakot, Pakistan; ^4^Department of Microbial Ecology, Groningen Institute for Evolutionary Life Sciences, University of GroningenGroningen, Netherlands

**Keywords:** PGPR, *Azospirillum*, potato, N_2_-fixation, IAA, TEM, plant inoculation

## Abstract

Rhizosphere engineering with beneficial plant growth promoting bacteria offers great promise for sustainable crop yield. Potato is an important food commodity that needs large inputs of nitrogen and phosphorus fertilizers. To overcome high fertilizer demand (especially nitrogen), five bacteria, i.e., *Azospirillum* sp. TN10, *Agrobacterium* sp. TN14, *Pseudomonas* sp. TN36, *Enterobacter* sp. TN38 and *Rhizobium* sp. TN42 were isolated from the potato rhizosphere on nitrogen-free malate medium and identified based on their 16S *rRNA* gene sequences. Three strains, i.e., TN10, TN38, and TN42 showed nitrogen fixation (92.67–134.54 nmol h^-1^mg^-1^ protein), while all showed the production of indole-3-acetic acid (IAA), which was significantly increased by the addition of L-tryptophan. *Azospirillum* sp. TN10 produced the highest amount of IAA, as measured by spectrophotometry (312.14 μg mL^-1^) and HPLC (18.3 μg mL^-1^). Inoculation with these bacteria under axenic conditions resulted in differential growth responses of potato. *Azospirillum* sp. TN10 incited the highest increase in potato fresh and dry weight over control plants, along with increased N contents of shoot and roots. All strains were able to colonize and maintain their population densities in the potato rhizosphere for up to 60 days, with *Azospirillum* sp. and *Rhizobium* sp. showing the highest survival. Plant root colonization potential was analyzed by transmission electron microscopy of root sections inoculated with *Azospirillum* sp. TN10. Of the five test strains, *Azospirillum* sp. TN10 has the greatest potential to increase the growth and nitrogen uptake of potato. Hence, it is suggested as a good candidate for the production of potato biofertilizer for integrated nutrient management.

## Introduction

Potato (*Solanum tuberosum* L.), of the family *Solanaceae*, is the third most important food crop in the world after rice and wheat. More than a billion people worldwide consume potato, and global crop production exceeds 350 million metric tons (Food and Agriculture Organization, 2014). It is a high fertilizer-demanding crop, which requires 250 kg ha^-1^ of nitrogen and 150 kg ha^-1^ of phosphorus to get an optimum yield (George and Ed, 2011). These requirements not only increase the cost of production but also cause severe environmental problems (David et al., 2002). A more ecologically friendly and economical approach to this problem may lie in the exploitation of the rhizosphere microbiome. Soil microbes constitute a largely unexplored biochemical wealth which have a profound role in biogeochemical cycles and may directly or indirectly impact on the nutrient status of soil (Khosro and Yousef, 2012).

Rhizobacteria are plant-associated bacteria that are able to colonize and persist in the vicinity of, on, or inside the roots of plants. Due to the often raised nutritional concentrations and root exudates, plant roots serve as ‘microbial hot spots’ in the soil. The rhizobacteria can colonize different sites of the rhizosphere (e.g., the root surface or interior) and directly or indirectly stimulate plant growth. When introduced to seeds, roots or into the soil, these bacteria, which include plant-growth- promoting rhizobacteria (PGPR) can solubilize insoluble phosphates, produce plant growth hormones, convert atmospheric nitrogen to ammonia or suppress the growth of plant pathogens (Pérez-Montaño et al., 2013). *Alcaligenes, Agrobacterium*, *Arthrobacter*, *Azospirillum, Azotobacter*, *Bacillus*, *Bradyrhizobium*, *Burkholderia*, *Enterobacter, Frankia, Klebsiella*, *Pseudomonas, Rhizobium*, and *Serratia* are genera that encompass common PGPR with known benefits on different crop plants (Bhattacharyya and Jha, 2012; Tailor and Joshi, 2014). Research on PGPR has been increasing and a number of experiments, both *in vitro* and *in vivo*, have been carried out on different crops. This has included wheat (Majeed et al., 2015), rice (Lucas et al., 2009), maize (Qaisrani et al., 2014), soybean (Cassán et al., 2009), sunflower (Shahid et al., 2012), and bean (Pérez-Montaño et al., 2013). These studies have shown the potential of PGPR to foster the growth and yield of such crops, with minor inputs of agrochemicals and lowered consequences for the environment.

The rhizosphere engineering of potato using PGPR still leaves a lot to be desired. Limited data are as yet available with respect to PGPR colonization, disease suppression (Andreote et al., 2009; Ghyselinck et al., 2013) and growth promotion (Mohammed et al., 2013) in potato. The reported PGPR from potato include mostly *Bacillus* and *Pseudomonas* sp. that have been used for improving phosphorus uptake (Hanif et al., 2015), production of indole acetic acid (IAA) and biocontrol activity (Calvo et al., 2010; Hunziker et al., 2015) and induced systemic resistance (Ardanov et al., 2011). *Rhizobium*-induced systemic resistance has also been reported in potato (Reitz et al., 2001). Although *Azospirillum* sp. have been reported to be associated with the rhizosphere of a number of crops, up till now no data are available with respect to the association of this genus with potato.

We have isolated bacteria from potato growing areas of central Punjab, Pakistan. These areas are geographically heterogeneous and share up to 80% of the total potato production of the country. It was hypothesized that the rhizosphere microbiome of these sites may contain a variety of potential PGPR that can augment plant growth especially potato. With this aim, taxonomically diverse bacteria were obtained and a comparative study was conducted for their functional characterization, potato tuber inoculation response and nitrogen uptake. We describe the potential of *Azospirillum* for growth promotion of potato. To the best of our knowledge, this is the first report of *Azospirillum* association with potato roots.

## Materials and Methods

### Soil Sampling and Bacterial Isolations

Potato rhizospheric soil samples were collected from two different areas of Punjab, Pakistan, i.e., Jhang (N: 31° 13.712′, E: 072° 16.080′, 134 m above sea level) and Sahiwal (N: 30° 37.707′ E: 73° 01.203′, 157 m above sea level). Samples were immediately placed on dry ice and transferred to the laboratory for further work. Nitrogen-free malate (NFM) semi-solid medium was used for the isolation of diazotrophic bacteria (Okon et al., 1977). Potato root pieces along with rhizosphere soil were introduced into 5 mL of NFM medium in glass vials. After 48 h of incubation at 28 ± 2°C, 100 μL from each vial were transferred to a fresh vial containing semi-solid NFM. For enrichment, this procedure was repeated at least six times and then a loopful of culture was streaked on NFM agar plates. Single colonies were picked from these plates, streaked and purified by repeated sub-culturing on fresh Luria Bertani (LB) agar plates. The purified isolates were maintained on LB agar plates for further studies. Isolates were stored in 20% (v/v) glycerol at -80°C.

### Morphological Characterization

Colony morphology of purified bacterial isolates was studied by streaking on LB agar plates, followed by incubation of the plates at 28 ± 2°C for 24 h. The cell shape and motility of the bacterial strains were observed using light microscopy (Nikon LABOPHOTO-2, Japan). Gram staining was done as described by Vincent (1970).

### Phenotypic Microarray

The metabolic potential of the *Azospirillum* sp. TN10 was evaluated by using BIOLOG GN2 microplates (Müller and Ehlers, 2007). Strain TN10 was grown on LB agar plates for 48 h at 28 ± 2°C. It was then harvested in 1.5 mL Eppendorf tubes containing 1 mL of DEPC water and starved for 3 h. The culture was then mixed with inoculation fluid IF-0a and a redox indicator as per the manufacturer’s instruction. 100 μL were added to each of the 96 wells in the carbon utilization plate, PM2A (Biolog, Hayward, CA, USA). The plates were incubated at 28 ± 2°C for 24 h and observed on a VERSA max micro-plate reader (Molecular Devices, USA) with softmax pro-software for qualitative and quantitative analysis (Line et al., 2011).

### Identification and Phylogenetic Analysis of Bacteria Based on 16S rRNA Gene Sequence

Genomic DNA was extracted using the UltraClean Microbial DNA Isolation Kit (Mo Bio Laboratories, Inc., New York) according to the manufacturer’s instructions. The 16S rRNA gene was amplified with primers fD1 and rD1 (Weisburg et al., 1991), using conditions described by Shahid et al. (2012) in a thermal cycler (PeQLab, advanced Primus 96). Purified PCR products were sequenced commercially (Eurofins, Germany). The sequenced products were analyzed using sequence scanner software package. Both ends were joined by Cap 3 assembly software and examined by NCBI BLAST (http://blast.ncbi.nlm.nih.gov/Blast.cgi) against the GenBank database. Multiple sequence alignment was performed and phylogeny was determined by neighbor-joining using MEGA6 software package (Tamura et al., 2013).

### Quantification of Nitrogen Fixation

Bacterial isolates were assessed for their nitrogen fixing ability using the acetylene reduction assay (ARA) as described by Hardy et al. (1968). Each isolate was grown in NFM semisolid medium for 72 h at 28 ± 2°C and evaluated for nitrogenase activity by gas chromatography (Thermoquest, Trace GC, Model K, Rodon Milan, Italy) using a Porapak N column and flame ionization detector (FID), following the standard protocol described by Park et al. (2005). The nitrogenase activity of the isolates was expressed as nmoles of ethylene formed per h per mg of protein.

### Production of Indole-3-Acetic Acid (IAA)

Indole acetic acid production ability was determined by colorimetric analysis and HPLC. The test was performed both in the presence and absence of L-tryptophan as precursor of IAA. One hundred mL LB broth in 250 ml Erlenmeyer flasks was inoculated with 100 μl of overnight bacterial culture, adjusted to optical density 0.6 (10^7^–10^8^ CFU ml^-1^) measured at 600 nm (Camspec M350 double beam UV Visible, UK). The bacteria were grown at 28 ± 2°C for 72 h with continuous shaking at 150 rpm. Supernatant was collected by centrifuging at 4000 × *g* for 15 min. Half of the supernatant (≈50 ml) was filtered through 0.2 μm nylon filters (Millipore, USA). IAA was detected by mixing 100 μL of Salkowski reagent (1 ml 0.5 M FeCl_3_, 30 ml concentrated H_2_SO_4_ and 50 ml distilled H_2_O) with 100 μL of filtered supernatant and allowed to react at room temperature for 20 min. IAA production was confirmed by pink color development as quantified at 540 nm (Camspec M350 double beam UV Visible, UK). A standard graph was prepared against concentrations 10, 20, 30, 40, 50, and 100 ppm IAA (Sachdev et al., 2009). Blanks (LB broth medium and Salkowski reagent) showing yellow color were below the detection limit. The remaining half of the supernatant (≈50 ml) was acidified to pH 2.8 with 1 N hydrochloric acid and extracted three times with equal volumes of ethyl acetate (Tien et al., 1979). The extract was analyzed on HPLC (Perkin Elmer, USA) fitted with C-18 column, using methanol (30:70 v/v) mobile phase at a flow rate of 0.5 mL min^-1^ as described (Shahid et al., 2014).

### Analysis of *nifH* Gene

Nested PCR was used for *nifH* gene amplification following Pereira e Silva et al. (2011). The first PCR was carried out with primers FPGH19, 5′-TACGGCAARGGTGGNATHG-3′; (Simonet et al., 1991) and PolR, 5′-ATSGCCATCATYTCRCCGGA-3′ (Poly et al., 2001) that resulted in a product of 490 bp. Two μL of this first PCR product were used as the template in the second reaction with primers PolF, 5′-TGCGAYCCSAARGCBGACTC-3′ and AQER, 5′-GACGATGATYTCCTG-3′ (Poly et al., 2001). To confirm amplification of the desired PCR product, amplicons were run on 1.5% agarose gels in TAE buffer (20 mM Tris, 10 mM acetate, 0.5 mM EDTA; pH 8.0) along with a 1-kb DNA ladder (Promega, Leiden, the Netherlands). PCR products were purified using the QIAquick PCR purification kit (Qiagen, USA) and sent to Macrogen (Seoul, Korea) for sequencing. The sequenced product was analyzed in the same way as described for the 16S rRNA gene. The Molecular Modeling Database (MMDB) from NCBI combined with the software Cn3D (NCBI) was used to model the 3D structure of the nitrogenase encoded by the *nifH* gene.

### Plant Inoculation Experiments

The bacterial strains were grown in 100 mL of LB for 24 h at 28 ± 2°C. After growth, cells were harvested by centrifugation at 4000 × *g* for 20 min at 4°C and resuspended in 100 mL of sterile saline (0.89% w/v NaCl in water). In order to evaluate plant growth promotion activities, medium-sized (2–3 cm) tubers of potato (variety Kuroda) were used. Potato tubers were thus surface-sterilized with sodium hypochlorite (10% v/v) for 8 min followed by extensive washing with sterilized distilled water. Sand was also sterilized by soaking in 0.5 N nitric acid for 24 h, washed thoroughly to remove acid, dried and then autoclaved.

There were five bacterial inoculation treatments along with one uninoculated control treatment, each with four replicates. Single tubers, with more than two eye buds, were dipped in the inoculum (containing ≈10^9^ CFU mL^-1^) for 15 min and then placed in pots containing 600 g of sand, after which pots were placed in a growth room (16/8 h light/dark and 20/8°C day/night temperature). A second inoculation was done at day 15 after seedling emergence, by mixing inocula in half-strength Hoagland solution (Arnon and Hoagland, 1940) at rates of 2 mL per pot. Plants were irrigated with Hoagland solution and sterilized water (1:1) every 48 h (150 mL per pot). Plants were harvested after 60 days and measurements were made of shoot and root length, and fresh and dry weights. Colonization by inoculant strains was evaluated by recovering cells from the respective rhizospheres on LB plates. Careful uprooting of the potato plants with intact roots was used, followed by gentle shaking in sterile distilled water to remove the loosely adhered sand. Bacteria were recovered by dilution plating technique using 1 g of strongly adhering sand in 9 mL sterile water (Seldin et al., 1998).

### Ultrastructure Studies

Roots from the above experiment were washed with sterile distilled water to remove the sand particles. They were then cut into pieces (∼1–3 cm) and embedded in water agar, followed by cutting out ∼2–3 mm agar cubes. The cubes were put in 1.5 mL tubes in the presence of 5% gluteraldehyde as a fixative for 16–18 h, after which the fixative was replaced with 0.2 M PIPES buffer (Salema and Brandao, 1973). After washing twice for 1 h with fresh buffer, samples were treated with 0.2% osmium tetraoxide, dissolved in PIPES buffer for 16–18 h and again washed twice for 30 min with sterile distilled water. After being treated with 5% aqueous uranyl acetate for 16–18 h, the samples were washed twice with sterile distilled water for 30 min. The samples were then immersed twice in absolute ethanol for 30 min, followed by immersion in propylene oxide (100%) for 30 min for dehydration. Infiltration of samples was carried out with propylene oxide at a ratio of 1:1 for 24–48 h and then with spur resin for a further 24–72 h, using benzyl dimethyl amine as the accelerator in all infiltration steps. The samples were polymerized for 72 h at 70°C on flat embedded molds, followed by incubation at room temperature for at least 24 h before cutting. Sections (150–200 nm) were cut using an ultra-microtome (RMC-7000; Boeckeler Instruments, USA) and carefully placed on copper grids. Sections were then double-stained with uranyl acetate (30 min) and lead citrate (10 min), washed with deionized water and observed under transmission electron microscope (TEM; JEOL JEM 1010, USA).

### Statistical Analyses

Data on different plant parameters of the pot experiment were analyzed statistically by analysis of variance (ANOVA; Steel et al., 1997) using STATISTIX 8.1 software (Tallahassee, FL, USA). Treatment means were compared by applying least significant difference tests (LSD) at 5% probability.

## Results

### Isolation, Characterization, and Identification of Rhizobacteria

Rhizospheric soil samples were collected from Jhang and Sahiwal, which are major potato growing areas of Punjab, Pakistan. Five bacterial strains were purified from enrichment culture on NFM semisolid medium. **Table 1** shows the morphological and physiological characteristics of these bacteria. Bacterial isolates TN10 and TN14 were obtained from Jhang and showed pink, round colonies with vibroid motile cells and convex, round colonies with motile rods, respectively. The other bacterial isolates, TN36, TN38 and TN42, were obtained from Sahiwal and showed round colonies which contained motile short rods, except TN42 which was non-motile. All isolates were Gram-negative. Bacterial isolate TN10 utilized 52 carbon sources out of 95 in the PM2A, BIOLOG microplate, showing a very diverse metabolic potential.

**Table 1:** Morphological characteristics of the bacterial isolates from potato and their identification on the basis of 16S *rRNA* gene sequence analysis.

Isolate code	Colony morphology	Cell morphology	ARA	Maximum similarity of 16S *rRNA* with (%)	GenBank accession no. of 16S rRNA gene
TN 10	Pink, round	Vibroid rods	134.54 ± 13.99	*Azospirillum* sp. FR667907 (99%)	LN614537
TN 14	Convex, round	Rods	-ve	*Agrobacterium* sp. JN628031 (99%)	LN614534
TN 36	Mucoid, round	Short rods	-ve	*Pseudomonas* sp. EF540467 (99%)	LN614533
TN 38	Creamy, smooth	Short rods	96.68 ± 7.61	*Enterobacter* sp. CP003678 (99%)	LN614535
TN 42	Milky, round	Rods	92.67 ± 8.42	*Rhizobium* sp. HG518323 (99%)	LN614536


*All strains were motile except TN42. ARA, acetylene reduction assay (nmol h^-*1*^mg^-*1*^ of protein). Values are averages of three independent replications. Standard errors of the means are mentioned between parentheses.*

**FIGURE 1:**
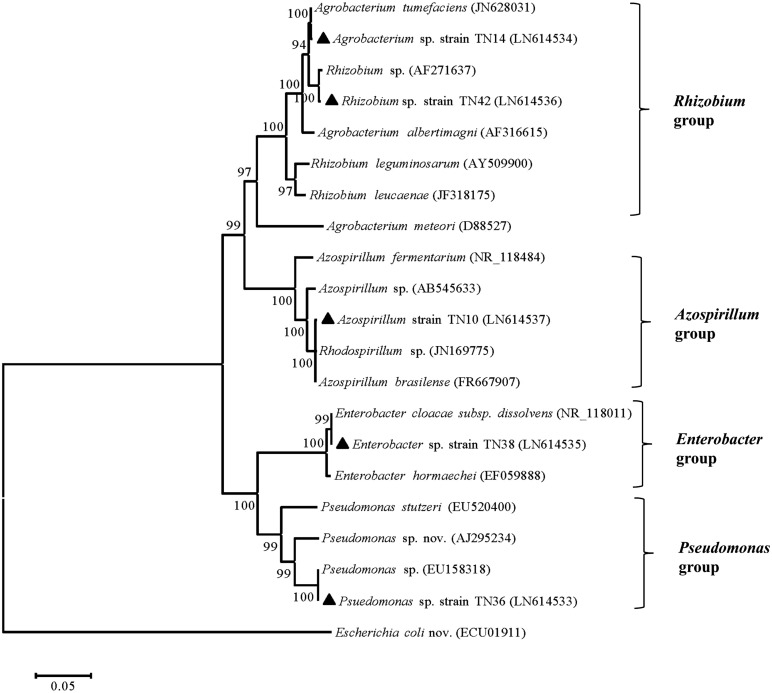
**Neighbor-joining tree showing the phylogenetic relationship of the PGPR strains isolated from the potato rhizosphere based on the sequences of the 16S rRNA gene (1100 bp).** Strains isolated in the current study “▲” along with those of closely related sequences obtained from GenBank. Numbers at the branching points are bootstrap values >90% (percentages of 1000 re-samplings). *Escherichia coli* served as the root. Bar shows sequence divergence of 0.05 nucleotides.

Based on 16S rRNA gene sequence analyses, bacterial isolate TN10 was presumptively identified as *Azospirillum* sp., TN14 as *Agrobacterium* sp., TN36 as *Pseudomonas* sp., TN38 as *Enterobacter* sp., while TN42 was a *Rhizobium* sp., all showing 99% homologies with the respective sequences in the GenBank database (**Table 1**). The 16S rRNA gene sequences were submitted to GenBank (nucleotide database) under the accession numbers LN614537, LN614534, LN614533, LN614535, and LN614536. Phylogenetic analyses using neighbor-joining showed two main clusters (α-proteobacteria and γ-proteobacteria) and four subclusters, with high bootstrap values (**Figure 1**). Strain TN14 and strain TN42 belong to *Rhizobium*, strain TN10 clustered in an *Azospirillum* clade, while strains TN38 and TN36 were affiliated with *Enterobacter* and *Pseudomonas*, respectively, at bootstrap values >95% with reference strains from the NCBI database (**Figure 1**).

### Nitrogenase Activity Measured by ARA and Phylogenetic Analysis of the *nifH* Gene

The ARA showed that *Azospirillum* sp. TN10, *Pseudomonas* sp. TN36, and *Rhizobium* sp. TN42 were positive for nitrogenase activity; strain TN10 showed the highest activity (**Table 1**).

**FIGURE 2:**
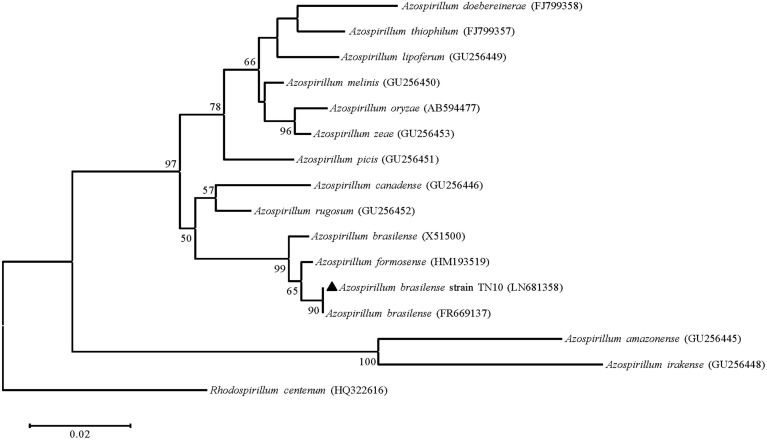
**Neighbor-joining phylogenetic tree based on *nifH* sequences (310 bp) of *Azospirillum* strains obtained from GenBank, including the *nifH* sequence of *Azospirillum* sp. strain TN10.** Numbers at the branching points are bootstrap values >50% (percentages of 1000 re-samplings). *Rhodospirillum* sp. served as the root. Bar shows sequence divergence of 0.02 nucleotides.

**FIGURE 3:**
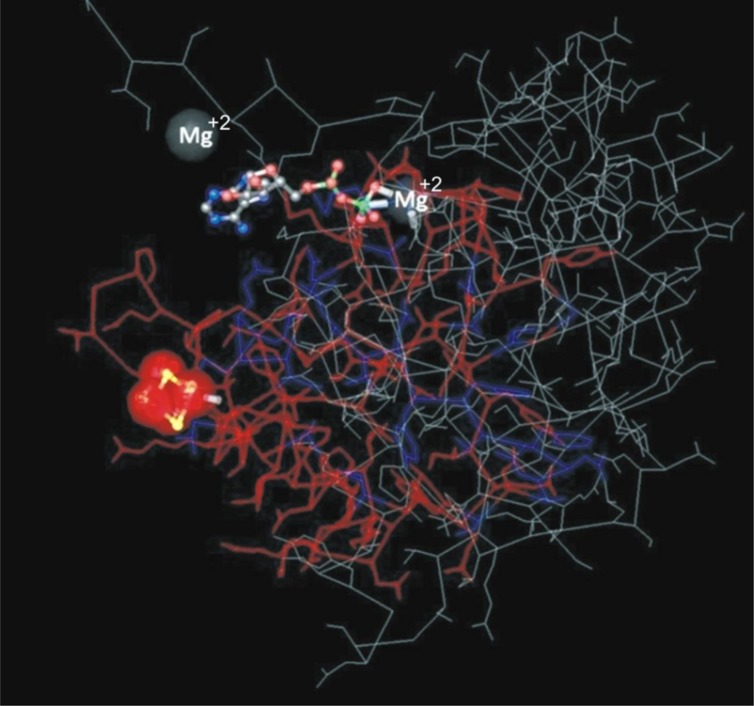
**Three-dimensional structure of Fe-nitrogenase protein of *Azospirillum* sp. strain TN10 based on 110 amino acids modeled using Cn3D software showing also the binding sites for Mg^2+^ which is suggestive of nitrogenase activity**.

Of three nitrogen-fixing strains, we could amplify the *nifH* gene only from *Azospirillum* sp. TN10. The 360 bp fragment was obtained and sequenced, after which the sequence was compared with GenBank database sequences. The sequence showed 99% similarity with an *Azospirillum brasilense* partial *nifH* gene, which encodes a nitrogenase iron protein (Acc. No. FR669137). Phylogenetic analysis based on neighbor-joining using the *nifH* gene sequence of strain TN10 and sequences from different *Azospirillum* genera showed that the *nifH* sequence of strain TN10 (LN681358) was affiliated with that of *A. brasilense* at a bootstrap value of 92 (**Figure 2**). The predicted 3D structure of the nitrogenase protein was 77% similar to a nitrogenase Fe-protein described for *Azotobacter vinelandii* (NCBI, PDB. Acc. No. 1RW4). This structure was modeled using MMDB from NCBI combined with the software Cn3D 4.3 (**Figure 3**).

### Quantification of IAA Produced by the Novel Bacterial Strains

**FIGURE 4:**
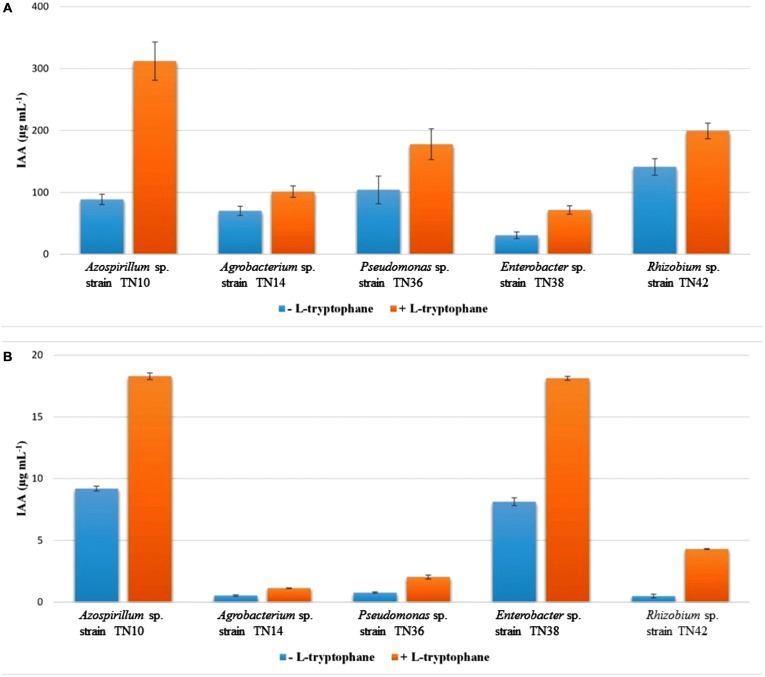
**Comparative quantification of IAA produced by bacterial strains isolated from potato using spectrophotometer **(A)** and HPLC **(B)**.** The values are means of three replicates. The standard errors of the means are represented as bars.

The Salkowski assay showed the production of IAA by all the bacterial strains with or without L-tryptophan induction. However, the amount of IAA was low in the absence of L-tryptophan (**Figure 4A**). *Enterobacter* sp. TN38 showed minimal IAA production (30.71 μg mL^-1^) while *Pseudomonas* sp. TN36 showed the highest (141.23 μg mL^-1^). Contrary to spectrophotometric detection, HPLC analysis (**Figure 4B**) showed low values of IAA produced by all five strains, with the highest detected in *Azospirillum* sp. TN10 (9.2 μg mL^-1^); this was found to increase with supplementation of L-tryptophan (18.3 μg mL^-1^; **Figure 4B**).

### Potato Root Colonization and Growth Promotion Potential of Bacterial Strains

**FIGURE 5:**
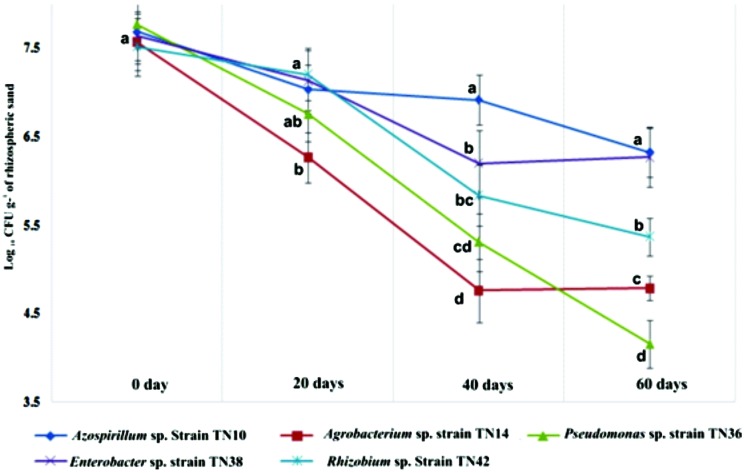
**Survival of different bacterial strains inoculated to potato (variety Kuroda) and population dynamics in the rhizosphere up to 60 days after inoculation.** The values are means of three replicates. The standard errors of the means are represented as bars. Comparison between treatments was carried out by one-way analysis of variance (ANOVA). The means followed by different letters (a, b, c) at one specific time (e.g., 20 or 40 days) are significantly different at the 5% level of significance.

The density of the bacterial populations in the potato rhizospheres changed in the different treatments at different growth stages of the plants. Bacterial populations were highest at the day of inoculation for all inoculants (>7.77 log CFU g^-1^ of rhizosphere sand). They then showed a continuous decline till day 60, except for the *Azospirillum* sp. TN10 and *Enterobacter* sp. TN38 strains, which maintained their population sizes at log 6.92 and 6.27 CFU g^-1^ of rhizospheric sand, respectively (**Figure 5**). No such bacterial populations were observed associated with the uninoculated plants.

**FIGURE 6:**
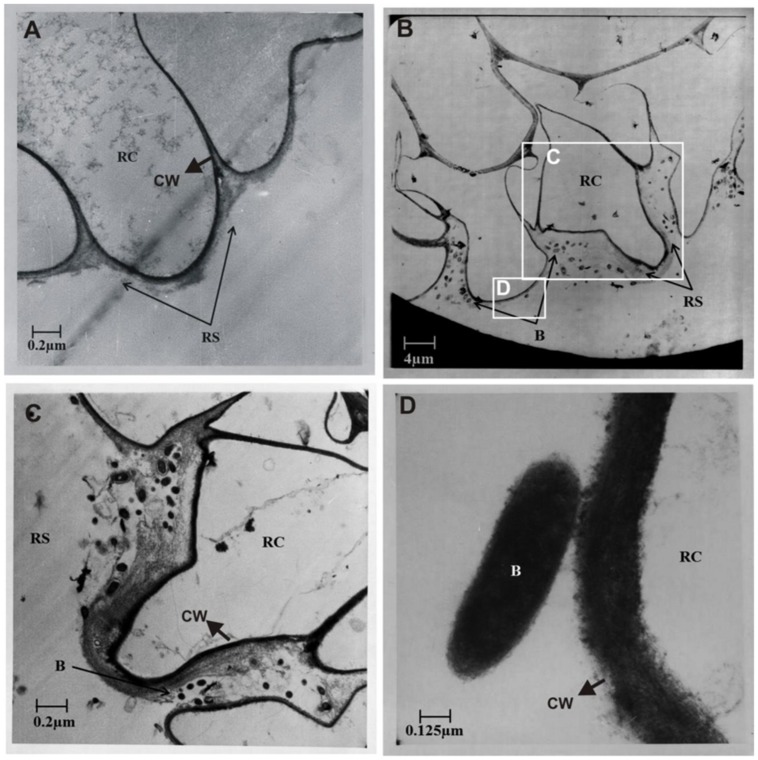
**Electron micrographs of *in vitro* grown root and rhizosphere sections of potato (variety Kuroda) from non-inoculated control plant **(A)** compared to plant roots inoculated with *Azospirillum* sp. strain TN10 **(B).** The bacterial attachment is evident on root surface within the rhizosphere and onto the cell wall.** **(C,D)** Are magnified micrographs from **(B)** marked with white box. RC, root cell; RS, rhizosphere; B, bacteria; CW, cell wall.

Ultrastructure studies further confirmed the colonization of potato roots by the *Azospirillum* sp. TN10 inoculant. No bacterial cells were observed in the uninoculated control plants (**Figure 6A**). In the strain TN10 inoculated roots, bacterial cells were found to be localized in the rhizosphere, in particular as attached cells on the root surface. However, no bacterial cells were observed inside the root cortical cells or the root hairs, showing that strain TN10 was not able to colonize the endosphere of potato (**Figures 6B–D**).

**FIGURE 7:**
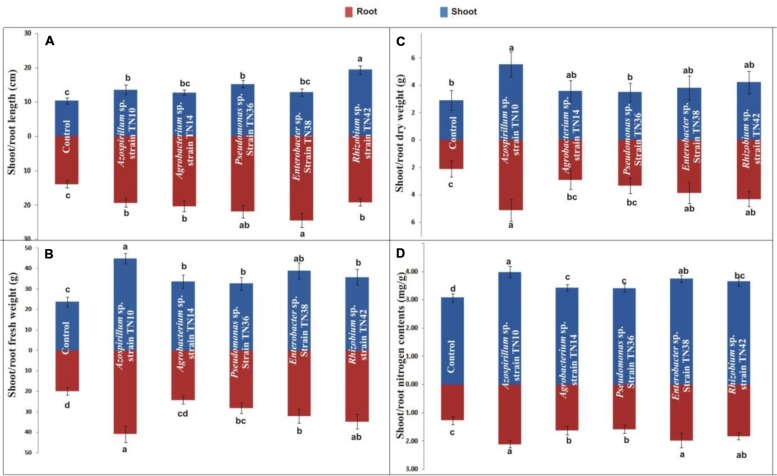
**Effect of bacterial inoculation on shoot and root length **(A)**, fresh weight **(B)**, dry weight **(C)**, and nitrogen contents **(D)** of potato (variety Kuroda) grown in sterilized sand in pots.** The values are means of three biological replicates. The standard errors of the means are represented as bars. Comparison between treatments was carried out by one-way analysis of variance (ANOVA). Values followed by different letters (a, b, c) are significantly different from each other at 5% level of significance.

**FIGURE 8:**
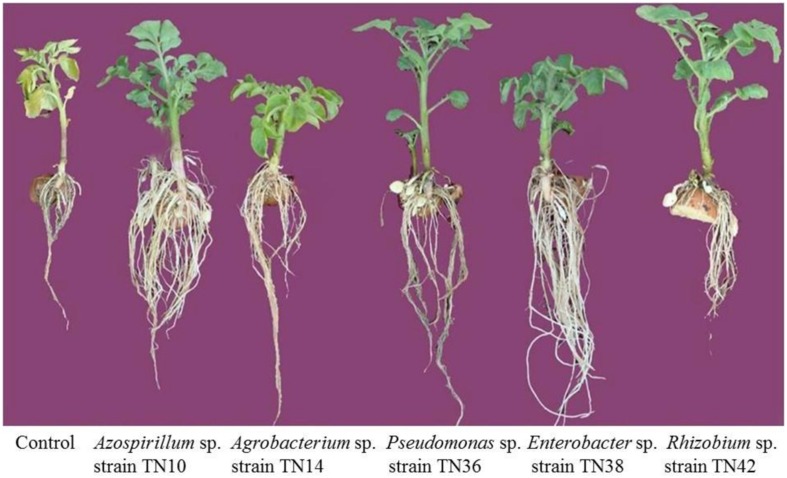
**Representation of potato plant health and root system as affected by bacterial inoculation of seed tubers (variety Kuroda).** Plants were harvested 60 days after sowing.

Overall, the inoculation with bacteria yielded significant positive effects on the plant growth parameters, including plant height, fresh and dry weight and total nitrogen levels (**Figures 7A–D**). Inoculation with *Rhizobium* sp. TN42 showed maximum increases in shoot length (85%), while inoculation with *Enterobacter* sp. TN38 showed maximum increases (76%) in root length (**Figures 7A** and **8**). Inoculation with *Azospirillum* strain TN10 resulted in maximum increases in shoot and root fresh and dry weights (**Figures 7B,C** and **8**). Specifically, this treatment showed ≈50% increases over the uninoculated control plants. Likewise, plants inoculated with *Azospirillum* sp. TN10 showed the highest N contents, both in the shoots and roots (3.98 and 2.1 mg g^-1^ dry wt). The uninoculated control plants showed the lowest N contents in both shoots and roots (**Figure 7D**).

## Discussion

The rhizosphere is a preferential niche for various types of microorganisms including a class of bacteria that are directly or indirectly beneficial for plant growth. The beneficial bacteria are designated as PGPR if they show different plant growth promoting properties, e.g., nitrogen fixation, phytohormone production, nutrient mobilization, or biocontrol abilities. The ultimate benefit of the use of PGPR is not only their plant growth promoting attributes, but also their environment friendliness and their cost-effective nature (Kaymak, 2011). Potato being an important food commodity needs special attention and requires extensive fertilization. The Sahiwal and Jhang regions are located in central Punjab, which is the prime area for potato production in Pakistan. Main crop rotations in Sahiwal include potato-maize-maize, with the potato varieties including Simply Red, Kuroda, Cardinal, and Rodio. In Jhang, main crop rotations are potato-maize-maize and potato-maize-fallow, with main potato varieties being Desiree, Cardinal, Kuroda, Mutta, and Sante. In both areas, about 250 Kg nitrogen and 150 Kg each of phosphorus and potash is used per hectare for potato production. To overcome the expensive fertilizer application and reduce costs for poor farmers, we isolated five bacterial strains from the potato rhizosphere using NFM medium. Identification based on 16S rRNA gene sequence analyses showed that these isolates, TN10, TN14, TN36, TN38, and TN42, belong to known PGPR genera, i.e.*, Azospirillum, Agrobacterium, Pseudomonas, Enterobacter*, and *Rhizobium*, respectively. Strain TN10 was identified as affiliated with *A. brasilense*, while for strains TN14, TN36, TN38, and TN42 species status could not be assigned. Phylogenetic analysis showed two main clusters, one encompassing α-proteobacteria (including *Agrobacterium*, *Rhizobium*, and *Azospirillum)* and the other encompassing γ-proteobacteria, including *Pseudomonas* and *Enterobacter*. The *Agrobacterium* and *Rhizobium* strains form one subgroup as both are closely related. In fact, *Agrobacterium* strain TN14 clustered with the previously reported *A. tumefaciens* JN628031 and *Rhizobium* strain TN42 with *Rhizobium* sp. AF271637. In addition, *A. brasilense* strain TN10 grouped in a separate subcluster with *A. brasilense* FR667907 and *Rhodospirillum* sp. JN169775. *Enterobacter* sp. TN38 and *Pseudomonas* sp. TN36 clustered with *E. cloacae* NR118011 and *Pseudomonas* sp. EU158318, respectively, both belonging to the γ-proteobacteria. Several members of these genera are well-documented for their plant growth promoting activities at different crops (Leyval and Berthelin, 1989; Mehnaz and Lazarovits, 2006; Afzal and Bano, 2008; Shahid et al., 2012; Qaisrani et al., 2014).

Biological nitrogen fixation can account for ≈97% of N input into unmanaged terrestrial ecosystems (Galloway et al., 2008). Nitrogen-fixing bacteria are usually isolated on NFM, which may yield false positive results. Thus bacteria isolated from N-free medium may not have the ability to fix nitrogen (Kuklinsky et al., 2004). Of the five strains isolated after repeated enrichment, only three, i.e., *Azospirillum* sp. TN10, *Enterobacter* sp. TN38 and *Rhizobium* sp. TN42, showed the ability to reduce acetylene to ethylene, while the *Agrobacterium* and *Pseudomonas* strains did not. Some of these N-free medium growing bacteria are efficient scavengers of traces of reduced nitrogen or they may be oligotrophs which grow on nitrogen fixed and released by other diazotrophs present in the mixed culture during the enrichment process (Tripathi et al., 2002). Phylogenetic analysis of the *nifH* gene of *A. brasilense* TN10 showed that it clustered with the *nifH* gene from *A. brasilense* FR669137. The 3D structure analysis revealed 77% similarities to the Fe-nitrogenase system of *A. vinelandii* showing also the binding sites for Mg^2+^, which is a requirement for nitrogenase activity along with dinitrogenase reductase, i.e., Fe-protein (**Figure 3**).

The effects of PGPR are often related to a manipulation of the network of plant hormones that are involved in growth and stimulation of root formation (Pérez-Montaño et al., 2013). Here, only the biosynthesis of IAA was evaluated. There are many pathways resulting in IAA production in rhizobacteria; some have tryptophan as a main precursor while others have tryptophan-independent pathways (Spaepen et al., 2007). Two main types of detection techniques are widely used for IAA quantification: (I) a colorimetric method using Salkowski reagents, (II) a high performance liquid chromatography (HPLC) method (Sachdev et al., 2009). Addition of tryptophan can affect the amount of IAA produced, because most bacteria have the tryptophan-dependent pathway. However, the amount of IAA produced varies across strains and also with the concentration of tryptophan added (Gutierrez et al., 2009). IAA production was determined in both tryptophan supplemented and non-supplemented medium by the aforementioned techniques. Addition of tryptophan increased IAA production in all strains, as shown by both assays, as reported earlier (Gutierrez et al., 2009). Contrary to the IAA concentrations observed by spectrophotometry, HPLC detected significantly lower concentrations in all strains. This may be attributed to the fact that Salkowski reagent can react with indole derivatives, thus giving false positive results (Kuang-Ren et al., 2003). Such indole derivatives have no role in plant growth, and hence the Salkowski assay is not adequate for the purpose of showing PGPR effects. It is useful, though, for screening large bacterial populations, as it is fast, thus reducing sample size, time and cost of eventual subsequent HPLC analyses.

Most strains exhibited multiple PGPR properties. These ultimately can provide benefits to plants in terms of stimulating growth. The variable innate PGPR potential of the strains may cause differential growth responses in plants (Ghyselinck et al., 2013; Qaisrani et al., 2014). We tested our bacterial strains only with respect to two traits, i.e., IAA production and nitrogenase activity. However, they might have potential for other PGPR traits which still need to be explored. In the plant inoculation assays, all bacterial strains improved plant growth as compared to the uninoculated control. Inoculation with *Azospirillum* strain TN10 exhibited maximum beneficial effects on plant growth, which we tentatively link to the ability of strain TN10 to fix nitrogen as well as produce high amounts of IAA. IAA can positively influence root elongation and lateral root development, which helps plants to acquire maximum water and essential nutrients. This may ultimately result in a well-established, vigorous and healthy plant. Bacterial strains producing phytohormones are known to influence the balance of plant phytohormones, eventually inducing different growth stages (Sturz, 1995) as well as, in an overall fashion, promoting plant growth (Ghyselinck et al., 2013). Greater plant dry weight production may also be ascribed to the enhanced leaf areas of the inoculated plants, especially the one inoculated with *Azospirillum* strain TN10, whereas greater shoot lengths reported may be positively correlated with greater photosynthetic rates and heightened transpiration efficiency because of availability of sufficient nitrogen, as compared to other plants (Byrd and May, 2000; Qaisrani et al., 2014). These results are consistent with those of earlier studies that show the effects of *Agrobacterium, Pseudomonas, Enterobacter* and *Rhizobium* on the growth of radish, French bean, sunflower, and rice, respectively (Ashrafuzzaman et al., 2009; Kaymak et al., 2009; Shahid et al., 2012).

The ability of bacterial inoculants to colonize plant tissue is an important factor to be addressed, because in the field the competition with other, indigenous, bacteria is tough. Here, all strains showed rhizosphere colonization and survival of up to 60 days. In particular, *Azospirillum* sp. strain TN10 showed high population densities and survival at the plant. In spite of the fact that the other strains showed a decline, apparently their population sizes up to 60 days were sufficient for the effects seen. Similar patterns of bacterial population declines were described by Fischer et al. (2010) in wheat. There may be several reasons for the fluctuations in bacterial population densities, with plant growth stage being a strong factor that affects the indigenous plant-associated communities in field-grown potato plants (Van Overbeek and Van Elsas, 2008). *Azospirillum* sp. strain TN10 showed relatively stable population sizes, which indicates it is a good root colonizer and PGPR in potato, as has been also reported for other crops (Steenhoudt and Vanderleyden, 2000; Guerrero-Molina et al., 2012; Qaisrani et al., 2014). Plant roots inoculated with *Azospirillum* sp. strain TN10, when examined by TEM, revealed strong bacterium-root associations. Adequate numbers of bacterial cells were thus present in the rhizosphere, and this may be related to the root area being rich in nutrients and having adequate micro-aerobic conditions that allow local nitrogen fixation to take place (Guerrero-Molina et al., 2012).

## Conclusion

This is a basic study of screening the PGPR associated with potato rhizosphere. We have demonstrated that among all the PGPR obtained, *Azospirillum* sp. strain TN10 due to its innate potential to produce plant growth hormone and convert atmospheric nitrogen to plant usable form has potential to increase potato growth and nitrogen uptake. This strain can be a potential candidate for production of potato biofertilizer for integrated nutrient management.

## Author Contributions

TN conducted experiments and wrote manuscript, SH supervised the study, AI helped in exp. and edited manuscript, MKH and AM helped in analysis, JDE helped in pot experiment.

## Conflict of Interest Statement

The authors declare that the research was conducted in the absence of any commercial or financial relationships that could be construed as a potential conflict of interest.
